# Evaluating the Potential of the Defatted By-Product of *Aurantiochytrium* sp. Industrial Cultivation as a Functional Food

**DOI:** 10.3390/foods10123058

**Published:** 2021-12-09

**Authors:** João Reboleira, Rafael Félix, Carina Félix, Marcelo M. R. de Melo, Carlos M. Silva, Jorge A. Saraiva, Narcisa M. Bandarra, Bárbara Teixeira, Rogério Mendes, Maria C. Paulo, Joana Coutinho, Marco F. L. Lemos

**Affiliations:** 1 MARE—Marine and Environmental Sciences Centre, ESTM, Politécnico de Leiria, 2520-641 Peniche, Portugal; joao.reboleira@ipleiria.pt (J.R.); rafael.felix@ipleiria.pt (R.F.); carina.r.felix@ipleiria.pt (C.F.); 2 CICECO—Aveiro Institute of Materials, Department of Chemistry, University of Aveiro, Campus Universitário de Santiago, 3810-193 Aveiro, Portugal; marcelo.melo@ua.pt (M.M.R.d.M.); carlos.manuel@ua.pt (C.M.S.); 3 LAQV-REQUIMTE, Department of Chemistry, University of Aveiro, 3810-193 Aveiro, Portugal; jorgesaraiva@ua.pt; 4 Division of Aquaculture and Upgrading, Portuguese Institute of the Sea and Atmosphere, Rua Alfredo Magalhães Ramalho, 6, 1495-006 Lisboa, Portugal; narcisa@ipma.pt (N.M.B.); barbara.p.b.teixeira@gmail.com (B.T.); rogerio@ipma.pt (R.M.); 5 CIIMAR, Interdisciplinary Centre of Marine and Environmental Research, University of Porto, Rua dos Bragas 289, 4050-123 Porto, Portugal; 6 Depsiextracta Tecnologias e Biológicas, Lda., Zona Industrial do Monte da Barca Rua H, Lote 62, 2100-057 Coruche, Portugal; mariacastelo@depsiextracta.eu (M.C.P.); joanacoutinho@depsiextracta.eu (J.C.)

**Keywords:** spent biomass, prebiotic potential, enzymatic digestion, biorefinery, circular economy, by-products

## Abstract

While *Aurantiochytrium* sp. is an increasingly popular source of polyunsaturated fatty acids (PUFAs), its extraction generates high amounts of waste, including the spent, defatted residue. The composition and bioactivities of this by-product could prove to be a major part of the sustainable valorisation of this organism within the framework of a circular economy. In this study, the defatted biomass of commercial *Aurantiochytrium* sp. was nutritionally characterised, and its amino acid profile was detailed. Additionally, the antioxidant and prebiotic potentials of an enzymatically digested sample of defatted *Aurantiochytrium* sp. were evaluated under a set of miniaturised in vitro assays. The nutritional profile of the spent *Aurantiochytrium* biomass revealed a protein and dietary-fibre rich product, with values reaching 26.7% and 31.0% for each, respectively. It also held high concentrations of glutamic and aspartic acid, as well as a favourable lysine/arginine ratio of 3.73. The digested samples demonstrated significant *Weissela cibaria* and *Bifidobacterium bifidum* growth-enhancing potential. Residual ferric reducing antioxidant power (FRAP) activity was likely attributed to antioxidant amino acids or peptides. The study demonstrated that some of the nutritional and functional potential that reside in the defatted *Aurantiochytrium* sp. waste encourages additional studies and the development of food supplements employing this resource’s by-products under a biorefinery framework.

## 1. Introduction

*Aurantiochytrium* sp. is a *Thraustochytrid* that has recently gained attention due to its high production of eicosapentaenoic acid and docosahexaenoic acid. They have emerged lately as an efficient economic alternative compared to other fish and microalgal oil sources by virtue of their simpler polyunsaturated fatty acids (PUFA) profiles and cost-effective culture conditions [1,2]. While the recovery of microbial oils avoids many of the problems associated with the traditional sources of PUFAs, the leftovers generated by such large-scale bioprocesses can still pose an environmental threat. A vast array of strategies and applications have been tested thus far in an attempt to add value to otherwise discarded microbial waste, with a large focus on the recycling of nutrients as a substrate for other economically feasible fermentations as well as the recovery of bioactive products [3,4,5]. Authors such as Medina (2015), Aida (2017), and Deshmukh (2021) have published distinct valorisation strategies applied to defatted microalgal or *Thraustochytrid* biomass, including the use as functional ingredients in biodegradable films, extraction of protein-rich antioxidant fractions, and direct use as nitrogen and phosphorous-rich additives to biofuel substrates [4,6,7]. The use of spent microalgal biomass has also been extended to livestock feed as either a soy or corn replacement as well as a nutritional supplement to traditional mixes [8].

Some of these applications fit under the designation of functional foods, which is a term that has acquired significant popularity in both social and scientific spheres. A recent definition given by Granato et al. (2020) states that functional foods, when regularly and efficaciously consumed as part of a diverse diet, can convey a positive effect on health beyond basic nutrition [9]. Said claims are regulated in most Western countries, limiting the classification to foods whose effects are verified via randomised, double-blind, and placebo-controlled clinical trials [10]. The potential held by functional foods in the prevention of many diseases deemed important in the 21st century, including obesity, type-2 diabetes, and several forms of cancer, has led to an enduring research interest over the course of the last 20 years [11,12,13]. The discovery of new functional foods is a large part of this effort, both as a way to find more bioactive ingredients and as a means to exploit new food resources in the form of new, high added-value products [13].

Due to the ever-growing demand for food of an expanding human population and their presence in an underexploited environment, marine food resources have found themselves under increased demand over recent years [14]. Among these, microalgae have confidently found their way into the niche market of food supplements. This was mostly due to exceptional amino-acid profiles in some cases comparable to terrestrial animal-sourced protein, as with the cyanobacteria *Arthrospira platensis* (commercial name “Spirulina”) and the green algae *Chlorella* [6,15]. High dietary fibre content is also a highly desired feature in certain types of functional foods. These long-chain polysaccharides are incapable of being digested by the human digestive process and have been linked to numerous gastrointestinal health benefits [16,17]. Certain types of dietary fibre can be fermented by the gut microbiota, selectively promoting the growth of beneficial *Bifidobacterium, Lactobacillus, Bacillus, Streptococcus, Saccharomyces* and *Lactococcus* strains [18]. In turn, the proliferation of these strains has been associated with improvements to gut health via the suppression of pathogenic bacteria, improved gastro-intestinal flow, and short-chain fatty acid-driven immunomodulation, which are factors that are currently deemed essential in preventing intestinal and colonic cancers [18,19]. Carbohydrates that can reach the caeco-colon undigested and demonstrate the microbiota-enhancing effects described fall under the designation of prebiotics [20].

Given the recognition of how important gut health is in the prevention of serious human disease, the study of prebiotics is currently at the height of its development [18]. The complexities of digestion and the changes it incurs on the chemistry and function of dietary compounds has led researchers to develop increasingly elaborate in vitro models when assessing the prebiotic potential of foods and supplements [19]. While these advances have greatly improved the authenticity of the attributed label of prebiotics and led to great new insights on the bioavailability of certain nutrients, they are often difficult to reproduce without highly specialised, often custom-made equipment or access to clinical samples of human faeces [18,21]. Thus, quick assessments of the prebiotic potential of new foods are difficult to execute with these methodologies. Simpler, in vitro batch fermentations are still considered valuable screening tools for this very reason, despite their inadequate simulation of the digestion process [17]. 

With the removal of its lipid content, the spent biomass of *Aurantiochytrium* sp. still holds the potential of a nutritionally and functionally valuable food product. Considering that these organisms can accumulate approximately 50% of their weight in lipids, the defatted and dried remainder is a highly concentrated mixture of proteins and carbohydrates of exotic origin and whose nutritional and functional potential remains unexplored [22]. The present study seeks to confirm these claims via chemical analysis of the spent *Aurantiochytrium* biomass in addition to a screening of its antioxidant and prebiotic potentials.

## 2. Materials and Methods

### 2.1. Recovery of Spent Aurantiochytrium sp. Biomass

*Aurantiochytrium* sp. biomass under the commercial label Algamac 3050 was purchased from Pacific Trading–Aquaculture Ltd. (Dublin, Ireland). The biomass was supplied in vacuum-sealed plastic bags and as coarse flakes approximately 1.5 mm in length and 0.5 mm in thickness. Removal of the lipid fraction was carried out to simulate its industrial processing for the recovery of PUFAs using a lab-scale Soxhlet extraction apparatus. Samples of 5 g of biomass were loaded in a Soxhlet cartridge and extracted with n-hexane for 6 h. At the end of the extraction, the spent biomass was recovered from the cartridge solvent and dried overnight in a 50 °C oven.

### 2.2. Chemical Analysis of the Spent Aurantiochytrium sp. Biomass

Protein content was estimated using a LECO FP-528 DSP nitrogen analyser (LECO, St. Joseph, MI, USA). Fat content of both whole and spent *Aurantiochytrium* was determined according using the Bligh and Dyer technique with the modifications employed by Burja et al. (2007) applied to the standard method [23]. Ash and fibre content were determined according to the Association of Official Analytical Chemists (AOAC) standard methods 942.05 and 985.29, respectively [24,25]. A protein-rich fraction obtained from the defatted *Aurantiochytrium* sp. was prepared according to the procedure detailed by Vallabha et al. (2016), with some modifications [26]. These included a more prolonged extraction time (overnight) and the combination of all precipitated protein fractions. These were analysed using reverse-phase high-performance liquid chromatography following the method of Bidlingmeyer et al. (1984) after being subjected to a 24 h acid hydrolysis with 6N HCl with 0.1% phenol under vacuum and derivatisation with phenyl thiocarbamoyl [27].

### 2.3. Enzymatic Digestion of the Spent Aurantiochytrium sp. Biomass

A two-step simulated digestion of the defatted *Aurantiochytrium* sp. biomass was performed with a procedure adapted from Gawlik-Dziki et al. (2009) [28]. A solution of simulated saliva was prepared by dissolving 2.38 g Na_2_HPO_4_, 0.19 g KH_2_PO_4_, and 8 g NaCl in 1 L of distilled water and adjusting its pH to 6.75. Then, the solution was supplemented with 200 U of α-amylase (EC 3.2.1.1.). The simulated gastric digestion solution was an acidic (pH 1.2) 0.32% pepsin (porcine stomach mucosa, pepsin A, EC 3.4.23.1) dilution in 0.03 M NaCl. In 50 mL plastic centrifuge tubes, an approximate weight of 10 g of defatted *Aurantiochytrium* sp. flakes were mixed with 50 mL of simulated saliva and incubated in a 37 °C water bath for 10 min with occasional stirring using a steel spatula. Then, the slurry was brought to a pH of 1.2 using 5 M HCl, after which 50 mL of the simulated gastric solution were added. Then, a 120 min, 37 °C water bath incubation took place with occasional manual stirring. Afterwards, the digestion was halted via a short exposure to a 70 °C water bath (approximately 60 s), and the pH was brought up to 6.0 with a 1 M solution of NaHCO_3_. Then, the entire digested slurry was treated as the digested defatted *Aurantiochytrium* sp. sample, from which 2 mL aliquots were gathered and stored at −20 °C prior to analysis.

### 2.4. In Vitro Prebiotic Potential Assay

**Figure 1:**
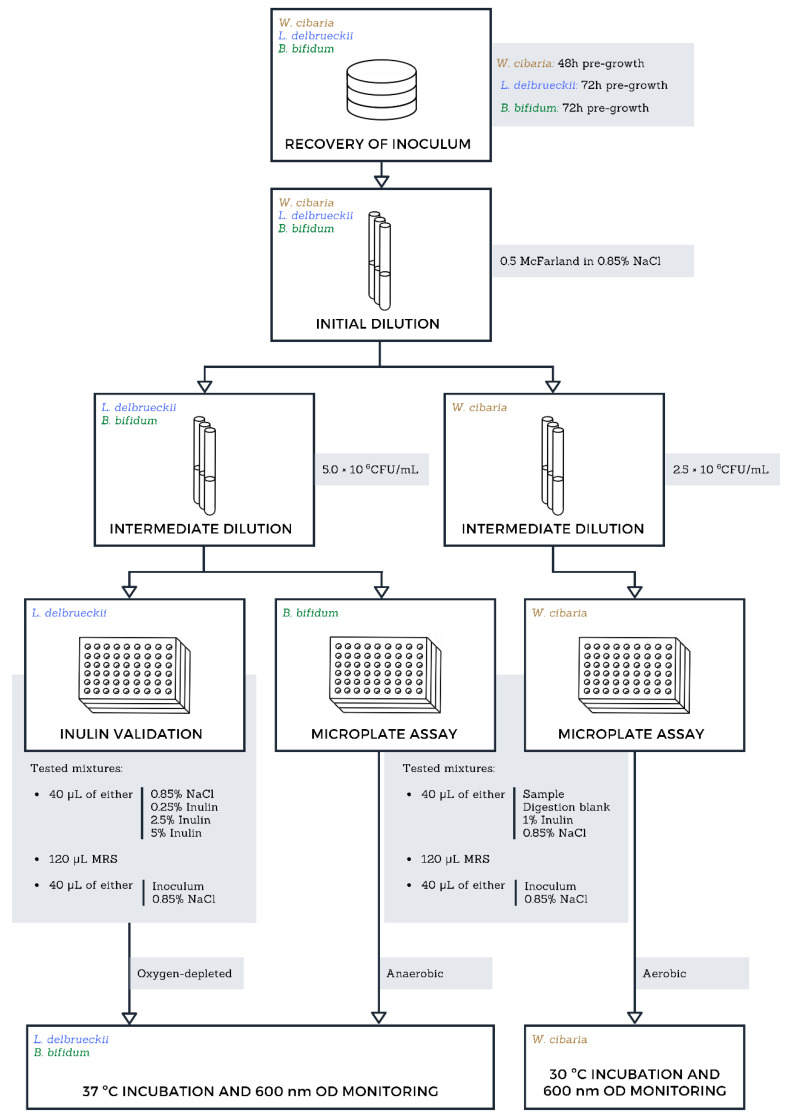
Flowchart representation of the miniaturised prebiotic potential assay employed in this study. Bacterial cultures were pre-grown in their respective optimal conditions for up to 72 h prior to the assay. The first step of the serial dilutions was performed identically for all cultures, with the following step adjusting for the required concentration. Inulin concentrations listed under “Tested mixtures” are higher than the tested concentrations as to account for the dilution occurring in the microplate well. Oxygen-depleted and anaerobic conditions were achieved using Merck’s Mini Anaerocult A and C kits following manufacturer specifications. The incubations were prolonged for up to 72 h.

The digested samples’ potential to promote the growth of probiotic lactic acid bacteria was evaluated using a simplified and miniaturised method based on publications of Wichienchot (2010) and Liu et al. (2016) and represented in Figure 1 [29,30]. The three bacterial strains selected for this assay include *Lactobacilus delbrueckii* subspecies *bulgaricus* DSMZ 20081, *Bifidobacterium bifidum* DSMZ 20456, and *Weissella cibaria* DSMZ 14295. Pre-cultures of *W. cibaria*, 48 h, 30 °C and pre-cultures of *B. bifidum* and *L. delbrueckii,* 72 h, 37 °C were prepared in MRS agar plates, with the latter two cultures having been maintained under anaerobic and oxygen-depleted atmospheres respectively using Mini Anaerocult A and C kits (Merck, Darmstadt, Germany). All pre-culture plates were prepared in triplicate as independent replicas. From these, 2.5 × 10^6^ CFU/mL (*W. cibaria*) and 5.0 × 10^6^ CFU/mL (*L. delbruecki* and *B. bifidum*) cellular suspensions were prepared in saline solution (0.85% NaCl; VWR). A set of master mixes were prepared encompassing all the necessary sample, blank, and control conditions in a 3:1:1 ratio of MRS medium, inoculum suspension, and sample, respectively. Digested *Aurantiochytrium *sp. samples were used without any further dilution, and digestion blanks provided a measure of growth induced by the enzymatic mixture (vehicle). Negative controls used the appropriate volume of saline solution instead of the digested sample. Then, 200 µL of each mixture were transferred to sterile round-bottom 96-well microplates and incubated for 72 h, with optical density (OD) readings occurring every 24 h at 600 nm using a microplate reader (EPOCH 2, BioTek Instruments, Winooski, VT, USA). Incubation of *W. cibaria* was conducted in aerobic conditions at 30 °C, while *B. bifidum* and *L. delbrueckii* microplates were enclosed in sealed bags under anaerobic and oxygen-depleted atmospheres at 37 °C as stated above.

A validation trial was performed using a 5.0 × 10^6^ CFU/mL suspension of *L. delbrueckii* and inulin as a reference probiotic. Inulin concentrations ranging from 0.01 to 1% (*w/v*) were prepared in master mixes with identical MRS media and inoculum ratios as the main assay.

### 2.5. Antioxidant and Lipid Oxidation Protective Assays

The digested and defatted *Aurantiochytrium* sp. sample was subjected to a set of three antioxidant potential assays. Ferric-reducing antioxidant potential (FRAP) activity assay was performed according to Dudonné et al. (2009) with slight modifications to sample dilution rates [31]. First, 195 µL of ferric 2, 4, 6-tri (2-pyridyl)-s-triazine (TPTZ) along with 5 µL of either sample or iron sulphate standard were incubated for 30 min at 30 °C. The concentrations of the latter ranged from 20 to 1000 µM. A minimum of three independent assays were performed for each extraction condition tested. The 2,2-diphenyl-1-picrylhydrazyl (DPPH) radical reduction assay used a 96-well microplate-adapted protocol [32,33]. The working reagent was prepared by dissolving DPPH radical in absolute ethanol at a concentration of 0.1 mg/mL. The assay was conducted by pipetting 10 µL of each standard’s or sample’s concentration and of the digestion vehicle as control per well (8 wells each). In four wells, 190 µL of working reagent was added, and in the other four, 190 µL of ethanol was added. The plate was incubated in the dark for 60 min at room temperature, after which its absorbance (Abs) was read at 515 nm (EPOCH 2 microplate reader, BioTek^®^ Instruments, Winooski, VT, USA). The amount of DPPH radical reduced by the standard per samples was calculated using a standard curve previously obtained, following the formula:

(1) $$\left[DPPH\right]\left(mM\right)=\frac{Abs_{Sample}-0.0391}{5.1238}.$$

For each sample, Abs (515 nm) was calculated by subtracting the mean absorbance of the wells containing the sample and ethanol to the mean absorbance of the wells containing the sample and working reagent. The assay was performed in triplicate. The lipid peroxidation inhibitory potential (LPIP) was evaluated by a method adapted from Félix et al. (2020) and Yen and Hsieh (1998) [32,33,34]. The method was designed based on the auto-oxidation of a pure suspension of PUFAs in contact with air, in which case the only peroxides present are the lipid peroxides, which are quantifiable by the thiocyanate method. Briefly, a linoleic acid (LA) suspension was prepared (20 mM LA in Tween 20 at 5.6 mg/mL prepared in phosphate-buffered saline (PBS) at 20 mM, pH 7.1) and used as substrate. Then, 25 µL of extract at 1 mg/mL were pipetted onto a 2.0 mL microtube, in triplicate, and 125 µL of LA suspension and 100 µL of PBS (same as above) were added. As blanks, tubes with extract but with LA suspension’s solvent (Tween 20 in PBS) instead of LA suspension were used (to determine peroxides native to the extract and subtract them from final result). As positive control (maximum peroxidation), 25 µL of extract vehicle (extract’s solvent) and 125 µL of LA suspension were used (along with 100 µL of PBS), and as negative control, 25 µL of vehicle as 125 µL of LA suspension’s solvent with 100 µL of PBS was used. All microtubes were incubated at 37 °C for 48 h in the dark and well capped. The experiment was performed in triplicate. Then, each tube was used to quantify peroxides by the thiocyanate method. From each tube, 20 µL (in triplicate) were sampled and added to a tube containing 940 µL of ethanol at 75% (v/v) and 20 µL of ammonium thiocyanate at 30% (w/v). Then, 20 µL of iron (II) chloride at 20 mM prepared in HCl at 3.5% (w/v) were added to each tube, and the mixture was properly homogenised using a vortex. Afterwards, each tube was used to read the absorbance in a microplate reader by pipetting 4 wells of 200 µL with the mixture. The absorbance was read at 500 nm, and the inhibitory potential was calculated:

(2) $$LPIP\;\left(\%\right)=100\frac{1-Abs_{SampleBlank}}{Abs_{Pos.Ctrl}-Abs_{Neg.Ctrl}}$$

### 2.6. Statistical Analysis

All experiments were performed with at least three replicas and are presented as mean ± standard error. All graphical representations, descriptive statistics, one-way analysis of variance (ANOVA), and multiple comparisons tests (Tukey’s honestly significant difference (HSD)) were all performed in GraphPad Prism v6.01 (GraphPad Software, Inc.; 2012, San Diego, CA, USA). Residual plots were used for all model assumptions, including normality, homoscedasticity, and independence. The type I error rate was at 0.05 for all statistical tests performed.

## 3. Results and Discussion

### 3.1. Chemical Analysis

Table 1 displays the macronutrient composition of both whole and defatted *Aurantiochytrium* sp. used in this study. A total lipid content of approximately 43% positions the samples used among the higher range of this parameter among other published results. Trovão et al. (2020) situated their set of *Aurantiochytrium* sp. within the 14 and 24% fat content range in their studies, while Ryu et al. (2013) achieved approximately 38.1% of lipidic weight growing *Aurantiochytrium* sp. in spent brewer’s yeast [35,36]. Regardless, percentages as high as the ones presented here were previously achieved [37]. The protein content is within the expected values for cultured *Aurantiochytrium* sp., with authors such as Sami et al. (2013) and Moran et al. (2019) reporting this parameter at around 15% [38,39]. Fibre content was found to be about 31% of its dry weight, which is a percentage that stands far higher than most published results for *Aurantiochytrium* sp. Moran et al., 2019 found a maximum fibre content of around 3.4% in *Aurantiochytrium limacinum*. Such high values are not common even in other thraustochytrids [22].

Looking at the macronutrient profile in isolation, *Aurantiochytrium* sp. in its defatted form already presents itself as a highly promising food product, with unusually high protein and dietary fibre contents. Similar profiles are found in marine organisms used as supplements, such as Spirulina and Chlorella [6]. With the loss of its lipid fraction, valued for its high PUFA content, the spent *Aurantiochytrium* sp. biomass may lose some of its nutritional richness. In turn, this depleted biomass is now much lighter in caloric content and thus much more compatible as a protein and dietary fibre supplement that is easily incorporated in a variety of diets.

### 3.2. Amino Acid Profile

The amino acid profile of the undigested spent *Aurantiochytrium* is shown in Table 2, listing the concentrations of 16 amino acids. Among these amino acids, eight essential amino acids were found. The major amino acids were glutamic acid (18 g/100 g) and aspartic acid (7.0 g/100 g), followed by serine, lysine, leucine, and proline. Cysteine, valine, and histidine were the among the least prevalent amino acids. The total amount of essential amino acids was higher than that of non-essential amino acids, with the high levels of leucine hinting at a potential use of this biomass in mid-workout energy snacks that aids muscle recovery and build-up [4]. While histidine levels were relatively low, they remain above the relative values of common plant or algae-based protein-rich supplements such as spirulina, soybean, and flaxseed. Histidine is a nutritionally essential amino acid that is also a precursor for several hormones (e.g., thyrotropin-releasing hormone), and critical metabolites affecting renal function, neurotransmission, gastric secretion, and the immune system [40]. The lysine/arginine/(Lys/Arg) ratio has been shown to positively affect the metabolic pathways of hypertension and have a positive effect on hypercholesterolemia, imparting lipidemic and atherogenic effects in rats even though the effects on humans were modest [26,41]. Although the exact mechanisms that lead to its positive effects are unknown, Yang et al. (2011) proposed that this amino acid ratio could limit the absorption rate of cholesterol [42]. The authors proposed either the slowdown of lipid absorption or promotion of 7 α-hydroxylase activity, which is a hepatic enzyme that limits the rate of cholesterol to bile acid conversion, as the mechanisms for this effect. In this study, the Lys/Arg ratio was found to be 3.73, which is quite similar to that of flaxseed (3.90) and a favourable ratio to be a useful protein ingredient in formulations intended to improve human health [40]. It should also be noted that the comparatively high amounts of glutamic and aspartic acid will likely contribute to an intensely umami flavour, which could grant any spent *Aurantiochytrium*-based supplement with desirable flavour-enhancing characteristics [43]. Such potential would need to be further investigated via sensory analysis.

### 3.3. Antioxidant and Lipid Protective Activities

Table 3 lists the results of the three antioxidant assays performed on the digested defatted *Aurantiochytrium* sp. sample. There was no lipid oxidation prevention demonstrable using the LPIP method, which is unsurprising given the nature of the spent *Aurantiochytrium* sample. The majority of bioactive compounds, including those with antioxidant potential, whose presence in *Aurantiochytrium* sp. was previously reported, are lipophilic carotenoids and sterols [45]. These are expected to be mostly absent from the defatted biomass. Any residual activity, such as that which was detected in the FRAP assay, was likely caused by amino acids containing sulphur side chains, such as cysteine and methionine, or aromatic side chains, such as tyrosine, phenylalanine, and tryptophan [46,47]. The contribution of these effects to the overall antioxidant activity is heightened by the lipid-removal process. DPPH radical reduction activity was, similarly to the LPIP assay, indicative of a lack of lipophilic antioxidant compounds. In this instance, the ethanolic nature of the reaction medium results in the precipitation of most protein, and thus, any amino acid-driven activity is unrepresented [47].

The presence of compounds with antioxidant activity in foods, regardless of their status as either functional or nutritious is, in most cases, highly desired. While the effects of dietary antioxidants in human health is still a contentious topic, their contribution as a positive factor in food preservation is generally well understood [48,49]. The presence of antioxidants in fatty foods, either as an ingredient or additive, is particularly desired, as these can greatly delay the loss in quality related to the oxidation of lipids [50]. While there are other compounds vulnerable to degradation under oxygen exposure, the defatted nature of the samples studied here means that lipid peroxidation phenomena are not as significant a concern for their long-term stability, and thus, a loss of antioxidant compounds may not be as sorely missed in a product based in defatted *Aurantiochytrium* [34]. Regardless, the presence of antioxidant proteins suggested by the FRAP activity assay could still provide a health benefit and warrants further research to determine their true chemical nature and concentrations.

### 3.4. Prebiotic Potential

#### 3.4.1. Method Validation

**Figure 2:**
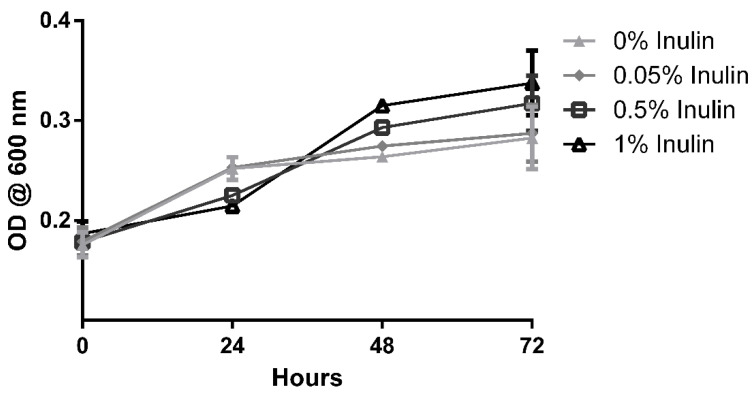
Growth of *Lactobacilus delbrueckii* under the effect of rising concentrations of inulin. Each point represents the average of at least three determinations ± standard error.

The results of the prebiotic potential method validation trial are shown in Figure 2. The assay was successful in demonstrating noticeable changes in growth both with and without the presence of additives in its media. While the higher concentrations of the reference probiotic led to higher optical density readings after 72 h of incubation, it is interesting to note that this trend was not constant throughout the intermediate measures. Growth readings after 24 h suggested a preference for lower or null concentrations of inulin, which is likely associated with a breach in its maximum tolerable presence for this organism. Prolonged incubation revealed a reverse trend, with the growth of organisms under higher concentrations overtaking those previously mentioned. It is possible that inulin concentrations somewhere above 0.05%, together with the tested concentration of MRS media, provided an overabundance of soluble sugars, resulting in unfavourable osmotic pressures. These stressors were eventually overcome by the organism, which then made use of the higher abundance of nutrients to surpass the growth in the other conditions. Since these dynamics were easily verified in the conditions tested here, the testing of probiotic growth effects was carried on using this method.

#### 3.4.2. Growth Effect on Probiotic Cultures

**Figure 3:**
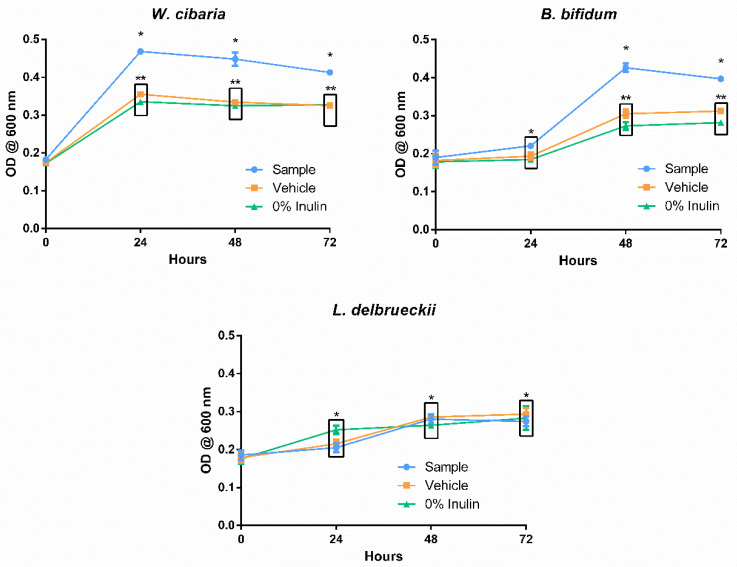
Growth of the probiotic strains *Bifidobacterium bifidum*, *Weissella cibaria,* and *Lactobacillus delbrueckii* under the presence of the digested defatted *Aurantiochytrium* sp. (Sample), the enzymatic digestion mixture (Vehicle), and the negative control saline solution (0% inulin). Each point represents the average of at least three determinations ± standard error. * and ** group measures are statistically identical within the same time point (either 24, 48, or 72 h).

Figure 3 shows the influence of the digested spent *Aurantiochytrium* solution (sample) and the digestion blank (vehicle) in the growth of *B. bifidum*, *W. cibaria*, and *L. delbrueckii* via a measure of optical density. For the first two microorganisms mentioned, the presence of the digested sample was favourable in promoting their growth, although the kinetics of each revealed differences that may influence the effectiveness of this sample as a prebiotic agent. The presence of digested defatted *Aurantiochytrium* solution had an immediate benefit on the growth of *W. cibaria*, as can be verified in the OD recorded after 24 h. This sudden growth appeared to consume nearly all the available substrate, and no further changes, worth noting, in cell density were observed over the course of the assay. *Weissela cibaria* has proven to be fastidious when compared to other lactic acid bacteria (LAB) [51]. Its accelerated growth kinetics and adaptability to harsh mediums has led to an increased interest in using it as a majority culture in sourdough starters [51]. *Bifidobacterium bifidum* revealed an equally favourable outcome for the probiotic growth enhancement potential of the digested defatted *Aurantiochytrium* solution, having reached 72 h of incubation with a significant DO difference between this sample and its digestion blank and growth control. In accordance with the features of a less fastidious anaerobic culture, the steepest increase in growth was observed between 24 and 48 h past medium inoculation, contrasting with the immediate spike in growth seen with *W. cibaria* immediately at 24 h. In contrast to the results commented so far, the growth of *L. delbrueckii* was unaffected by the presence of the digested defatted *Aurantiochytrium* sample or the digestion solution (vehicle). The scarcity of information regarding the non-lipid fraction of *Aurantiochytrium* sp. makes it difficult to point to specific causes for the selectivity of its prebiotic potential. Several studies testing the prebiotic potential of select compounds have also reported relatively lower growth of *L. delbrueckii*, but the authors did not establish likely causes [16,52,53]. Further studies of the non-lipidic fraction of *Aurantiochytrium* sp. might reveal hints about the selectivity of its prebiotic activity. 

The differences in kinetics between *W. cibaria* and *B. bifidum* are unlikely to reflect in a significant manner within in vivo conditions, given the extensive amount of biotransformations induced by digestion, as well as the influence of the remaining gut microbiota [19]. Selective or delayed growth of different gut bacteria is a hot topic of current-day prebiotic research, but the results shown here are more indicative of the differences between the growth characteristics of the studied strains rather than conclusive evidence of the selectivity of the substrate [54]. The results do show a significant increase in the growth of both *W. cibaria* and *B. bifidum* under the presence of digested spent *Aurantiochytrium* sp. Given the comparatively high amount of fibre content revealed by the chemical analysis, it is safe to say that this biomass, currently seen as industrial waste, may hold potential as a prebiotic supplement, with a stronger endorsement depending on further studies.

## 4. Conclusions

This study attempts an early exploration of functional food-related uses of spent *Aurantiochytrium* sp. biomass, which is a significant part of the industrial waste associated with the production of high added-value PUFAs from this source. Doing so could attribute this waste with value comparable to its lipid fraction and thus greatly increase the profitability and sustainability of all industrial exploitation of *Aurantiochytrium*. The chemical characterisations performed on the defatted biomass show a highly protein and dietary fibre-rich product that is simultaneously rid of most of its most caloric fraction. The amino acid profile revealed a fairly balanced distribution of essential variants, though it was not enough to warrant the use of this product as a protein supplement individually. High levels of glutamic and aspartic acid suggest it could be used as a source of flavour-enhancing umami compounds. The spent *Aurantiochytrium* was depleted of most of its lipophilic antioxidant compounds, but some residual activity was still registered by the FRAP assay, which was likely caused by antioxidant amino acids. The prebiotic potential assays revealed that after an in vitro enzymatic digestion, the sample held the capacity to enhance the growth of important probiotic strains. Together with its high dietary fibre content, these results point to a promising prebiotic supplement as one of the potential uses for the depleted biomass.

The results presented warrant a great deal of follow-up research, as many of its conclusions could be built upon a better understanding of the elemental composition of *Aurantiochytrium* sp., particularly of its non-lipid fractions. Additionally, the prebiotic potential demonstrated here is the result of a screening that is limited in scope and can be followed by either the testing of more strains and/or the use of a more in-depth digestion simulation and gut microbiota consortia from clinical samples. With these next steps fulfilled, a valuable application of the main by-products of *Aurantiochytrium* cultivation could be quickly implemented in both existing industries and upcoming biorefineries looking to maximise their profit and sustainability.

## Figures and Tables

**Table 1:** Macronutrient analysis performed on the whole *Aurantiochytrium* sp. (WA) and on its defatted counterpart (DA), shown as a percentage of dry weight. Each result is the average of at three measures ± standard error.

Sample ID	Lipid Content (g/100 g)	Protein Content (g/100 g)	Ash Content (g/100 g)	Fibre Content (g/100 g)
WA	42.7 ± 0.8	15.3 ± 0.7	10.7 ± 0.1	17.8 ± 1.5
DA	2.4 ± 0.8	26.7 ± 1.8	16.9 ± 0.9	31.0 ± 1.1

**Table 2:** Amino acid profile of the defatted *Aurantiochytrium* sp. biomass in g/100 g of extracted protein and as a percentage of total detected amino acids. A collection of comparable values reported in the literature was included for both *Aurantiochytrium* sp. and other *Thraustochytrids*.

Amino Acid	*Aurantiochytrium* sp. Spent Biomass (g/100 g)	% of Total AA	*Aurantiochytrium* sp. from Literature (g/100 g)		References (*Aurantiochytrium*)	Thraustochytrids from Literature (g/100 g)		References (Thraustochytrids)
			Min	Max		Min	Max	
Essential								
Alanine	2.2	4.0	0.8	3.9	[4,40]	0.53	7.5	[38,40,44]
Arginine	1.1	2.0	5.5	12.3		0.67	12.3	
Aspartic acid	7.0	12.7	5.6	7.1		2.91	14.7	
Glutamic acid	18	32.5	11.2	11.4		1.74	42.0	
Glycine	2.2	4.0	1.3	1.5		0.38	7.0	
Histidine	1.1	2.0	8.6	10.3		0.23	1.1	
Serine	4.2	7.6	2.6	3.2		0.46	10.8	
Threonine	1.8	3.3	0.3	0.8		0.38	1.5	
Non-essential								
Cysteine	0.79	1.4	0.3	0.4	[4,40]	0.14	1.4	[38,40,44]
Isoleucine	1.6	2.9	1.8	2.4		0.22	2.3	
Leucine	3.3	6.0	3.7	4.8		0.56	6.8	
Lysine	4.1	7.4	3.9	5.0		0.5	7.2	
Methionine	2.4	4.3	1.1	1.1		0.05	1.8	
Phenylalanine	1.4	2.5	2.1	2.7		0.36	3.7	
Proline	3.2	5.8	2.6	2.7		1.38	3.6	
Valine	0.93	1.7	2.7	3.4		0.34	4.0	

**Table 3:** Antioxidant potential of the post-digestion defatted *Aurantiochytrium* sp. biomass, according to the DPPH radical reduction potential, ferric-reducing antioxidant potential (FRAP), and lipid peroxidation inhibitory potential (LPIP) assays. Each result is the average of at least three measures ± standard error.

Sample ID	DPPH (mM DPPH/mL)	FRAP (Fe(II) eq (mM)/mL)	LPIP (% of Ctrl)
Digested DA	0.025 ± 0.022	152.5 ± 6.2	162.1 ± 6.2

## Data Availability

Data is available upon reasonable request.
